# Symmetrical Lupoid Onychodystrophy in Review: Lessons Learned From 31 Cases

**DOI:** 10.1111/vde.70114

**Published:** 2026-08-06

**Authors:** Jessica R. Calesso, Clarissa P. Souza, Stephanie R. Bruner, Adriane P. C. V. Bicalho

**Affiliations:** ^1^ Department of Veterinary Clinic and Surgery, College of Veterinary Medicine Universidade Federal de Minas Gerais Belo Horizonte Brazil; ^2^ Department of Veterinary Clinical Medicine, College of Veterinary Medicine University of Illinois at Urbana‐Champaign Urbana Illinois USA

**Keywords:** claw diseases, lupoid, onychodystrophy, onychomadesis

## Abstract

**Background:**

Symmetrical lupoid onychodystrophy (SLO) is a complex disease that exclusively affects the claws of dogs and represents a therapeutic challenge. Biopsy sampling of the third phalanx for histopathological evaluation has historically been the recommended diagnostic test, yet clinical diagnosis is currently encouraged. Numerous treatment protocols of varying success have been proposed for dogs with this condition.

**Objectives:**

The goals of this study are to report clinical findings, treatment protocols and outcomes of dogs with SLO, to propose a rationale for the clinical diagnosis and to discuss therapies for SLO.

**Animals:**

Thirty‐one dogs examined at a veterinary hospital from May 2004 to April 2024.

**Materials and Methods:**

Retrospective analysis of medical records from dogs diagnosed with SLO.

**Results:**

The average age of dogs at disease onset and diagnosis was 7 years old. Nineteen breeds were represented. Thirty dogs were clinically diagnosed with SLO, mostly presenting with onychodystrophy and onychomadesis, and in one a biopsy sample was collected. Different therapeutic combinations including tetracyclines alone or with niacinamide, pentoxifylline, essential fatty acids (EFAs), vitamin E, biotin and ciclosporin were used for the initial and/or maintenance treatment of SLO.

**Conclusions and Clinical Relevance:**

The distinctive presentation of SLO supports its clinical diagnosis. Medical treatment seems to support a favourable long‐term outcome, despite the wide variation in protocols and doses. EFAs were commonly used as initial and maintenance therapy, and tetracyclines could often be excluded from the treatment protocol, thus complying with good antimicrobial stewardship practice.

## Graphical abstract

**Background:** Symmetrical lupoid onychodystrophy (SLO) is a complex disease that exclusively affects the claws of dogs and represents a therapeutic challenge. Biopsy sampling of the third phalanx for histopathological evaluation has historically been the recommended diagnostic test, yet clinical diagnosis is currently encouraged. Numerous treatment protocols of varying success have been proposed for dogs with this condition. **Objectives:** The goals of this study are to report clinical findings, treatment protocols and outcome of dogs with SLO, to propose a rationale for the clinical diagnosis and to discuss therapies for SLO. **Conclusions and Clinical Relevance:** The distinctive presentation of SLO supports its clinical diagnosis. Medical treatment seems to support a favourable long‐term outcome, despite the wide variation in protocols and doses. EFAs were commonly used as initial and maintenance therapy, and tetracyclines could often be excluded from the treatment protocol, thus complying with good antimicrobial stewardship practice.

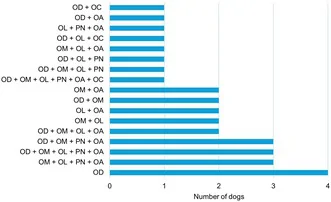

## 1 Introduction

Canine diseases affecting the claws are uncommon and can be frustrating conditions to diagnose and treat [1]. Symmetrical lupoid onychodystrophy (SLO) is a disease characterised by inflammation of the claw fold and abnormal claw formation that may eventually cause the claws to slough off. Initially, only a single claw may be affected, yet the disease will eventually progress to multiple claws on different paws within weeks to months after the initial presentation. Secondary bacterial infection may be associated with the condition, and affected dogs are often presented for evaluation of licking and chewing their paws, pain, acute lameness or suspected trauma causing claw avulsion. Regrowth of the claws, when they occur, is often abnormal, resulting in deformed, crumbling and brittle claws in otherwise healthy dogs [2, 3].

The pathogenesis of SLO is not completely elucidated and it is possible that various causes are involved with disease development, reflected by the diverse clinical presentation and response to treatments. Because of the increased risk reported in certain dog breeds such as German shepherd dogs [4], bearded collies [5], Gordon and English setters [6] and giant schnauzers [7], a genetic component is suspected. The clinical signs, the histopathological lupoid reaction and the genetic association between dog leucocyte antigens (DLA) class II and SLO provide support for the hypothesis of SLO being an immune‐mediated disease of the claw [7].

The first SLO reports established the recommendation for biopsy collection involving the amputation of the third phalanx or onychobiopsy collection without onychectomy for a definitive diagnosis [8, 9]. However, because there are very few diseases that cause claw abnormalities without other concurrent dermatological or systemic signs, and SLO histopathological findings are not pathognomonic, over the years referral clinicians have made a presumptive diagnosis of SLO based on history and clinical findings [2].

Treating SLO can be challenging although spontaneous remissions have been reported [3]. Different protocols and anecdotal doses are described and require long‐term owner compliance. Commonly used treatments include essential fatty acids (EFAs), tetracyclines (tetracycline or doxycycline or minocycline) and niacinamide, prednisone, pentoxifylline, azathioprine, ciclosporin, vitamin E, biotin and zinc supplementation [10].

There is limited updated information published about SLO forgoing digital biopsy sampling. Therefore, the objective of this retrospective study was to report signalment, clinical findings, treatment options and treatment outcomes of affected dogs seen at a tertiary hospital and to propose a rationale for the clinical diagnosis and management of SLO.

## 2 Materials and Methods

Medical records from the University of Illinois Veterinary Teaching Hospital between May 2004 and April 2024 were searched electronically using the key terms ‘symmetrical lupoid onychodystrophy’, ‘lupoid onychodystrophy’, ‘SLO’ and ‘lupoid’. All dogs' records mentioning an SLO diagnosis, with or without histopathological confirmation, were considered for the retrospective study.

The following data were collected from the medical records at initial presentation: sex, breed, weight, age at disease onset, age at diagnosis, clinical or histological diagnosis, secondary infection of the claw bed, number of paws affected, number of claws affected per paw, presence of claw abnormalities, concurrent cutaneous and/or systemic disease, strict diet trial, initial and maintenance therapies, follow‐up periods after diagnosis and response to medical treatment. The dogs were divided into four size categories based on weight: small (< 10 kg), medium (10–< 25 kg), large (25–< 40 kg) or giant (≥ 40 kg) [11].

Initial therapy was defined as the first medications and/or supplements prescribed at the time of diagnosis, and the response to treatment was evaluated at the first recheck. Maintenance therapy was defined as all medications prescribed or continued after rechecking. Response to initial therapy was recorded as ‘well‐controlled’ (when there was a marked improvement in clinical signs, as clinical remission was not expected to be achieved), ‘satisfactorily controlled’ (when clinical signs were partially improved, yet patients still showed discomfort) and ‘not controlled’ (when there was no clinical improvement). Response to maintenance therapy was classified as ‘controlled’ if clinical signs of SLO were static, and ‘uncontrolled’ if the patient experienced flare‐ups during the follow‐up period. Whenever the information was not recorded in medical records, the term ‘not available’ was assigned, and the data were not included in the descriptive analysis.

## 3 Results

The electronic search initially identified 67 medical records. Of these, 31 cases were enrolled in the study, and 36 were excluded owing to insufficient information to support the SLO diagnosis.

Eighteen of the 31 dogs (58.06%) were neutered males and 13 of 31 (41.93%) were spayed females. For the 14 dogs where the age at onset of clinical signs was available, the range was 1.9–13.7 years (average 7.1 years). The age at diagnosis in 30 of 31 dogs was in the range 1.2–13.8 years (average 7.4 years). Of the 31 dogs, 27 (87.10%) were pure‐bred, represented by 19 different breeds, and four (12.9%) were mixed‐breed dogs. German shepherd dog was the most affected breed (five), followed by Labrador retriever (three), great Dane (two) and Australian shepherd dog (two). Fifteen other breeds were represented by one dog each (Figure 1). Most dogs were giant or large (each 9 of 31; 29.04%) breeds, 8 of 31 (25.80%) were medium and 5 of 31 (16.12%) were small. The demographic data for each dog are found in Table S1 in the Supporting Information. Twenty‐one dogs (of 31, 67.74%) presented with concurrent diseases at the time of SLO diagnosis (Table S2).

**FIGURE 1:**
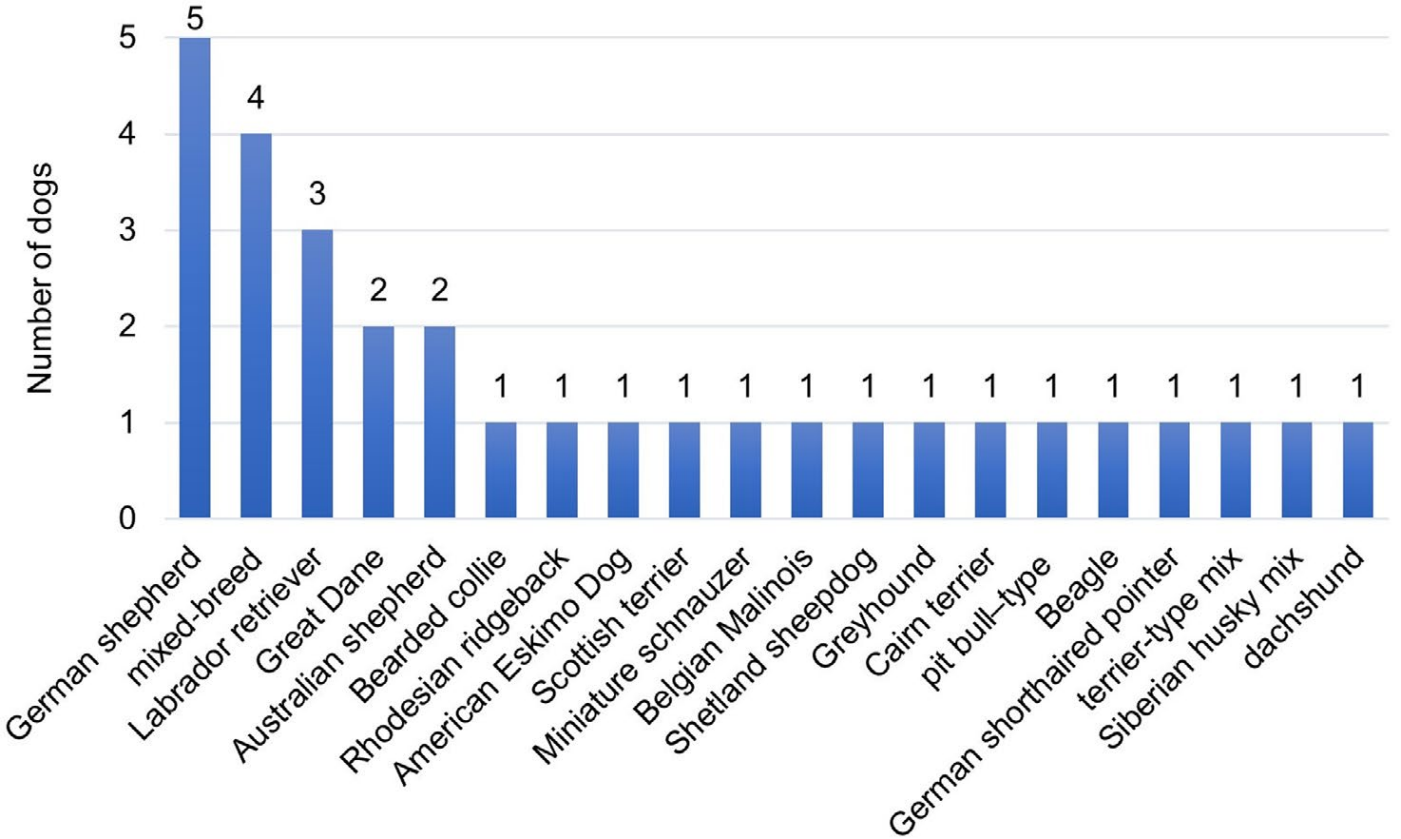
Breeds of dogs diagnosed with symmetrical lupoid onychodystrophy during the study period.

Clinical signs of claw abnormalities reported in medical records included paronychia (inflammation or infection of the claw fold), onychodystrophy (abnormal claw formation), onychomadesis (sloughing of the claw), onychalgia (claw pain), onycholysis (separation of the claw from the claw fold) and onychoschizia (splitting and/or lamination of the claws). Most dogs had more than one clinical finding (Figures 2 and 3; Table S3). Cytological evaluation was performed in 16 of 31 (51.6%) dogs with SLO and was negative for micro‐organisms in six of these (37.50%). Secondary bacterial and/or yeast infections in the claw fold were identified in 10 of 16 dogs (62.50%) (Table 1).

**FIGURE 2:**
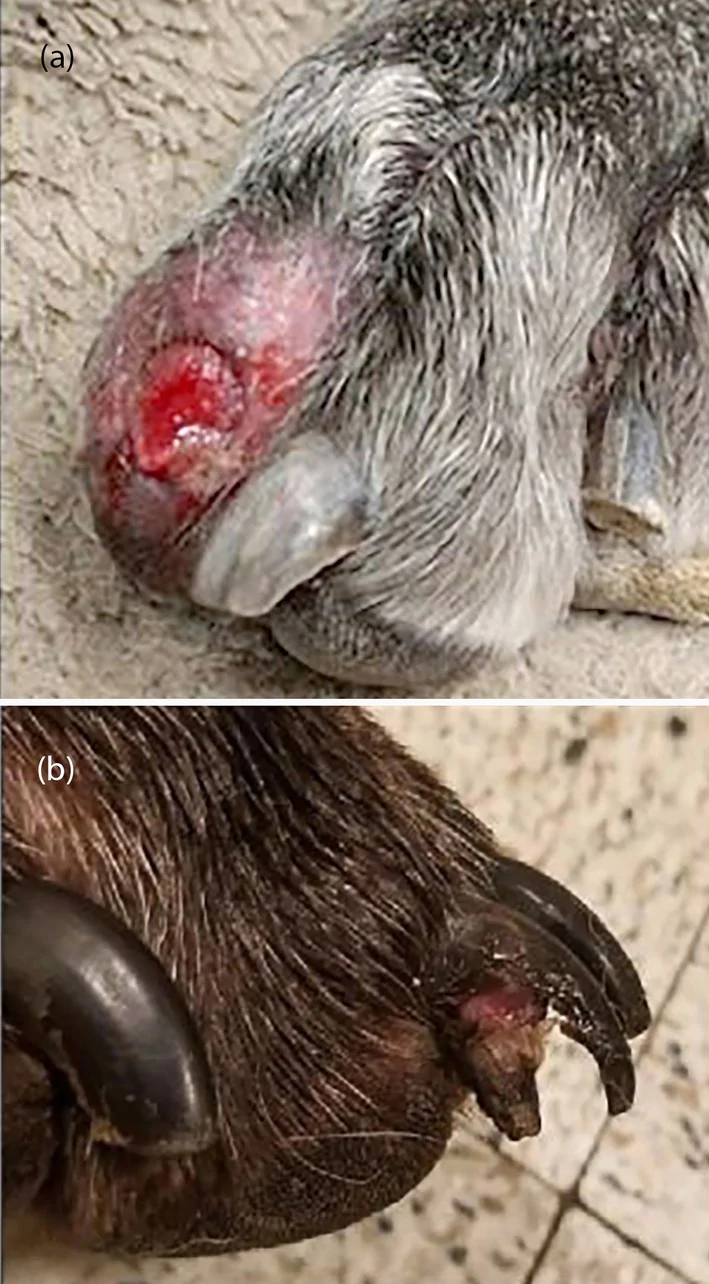
(a) Great Dane (Dog 6), presenting with paronychia and onycholysis. (b) German shorthaired pointer (Dog 31) presenting with paronychia and onychoschizia.

**FIGURE 3:**
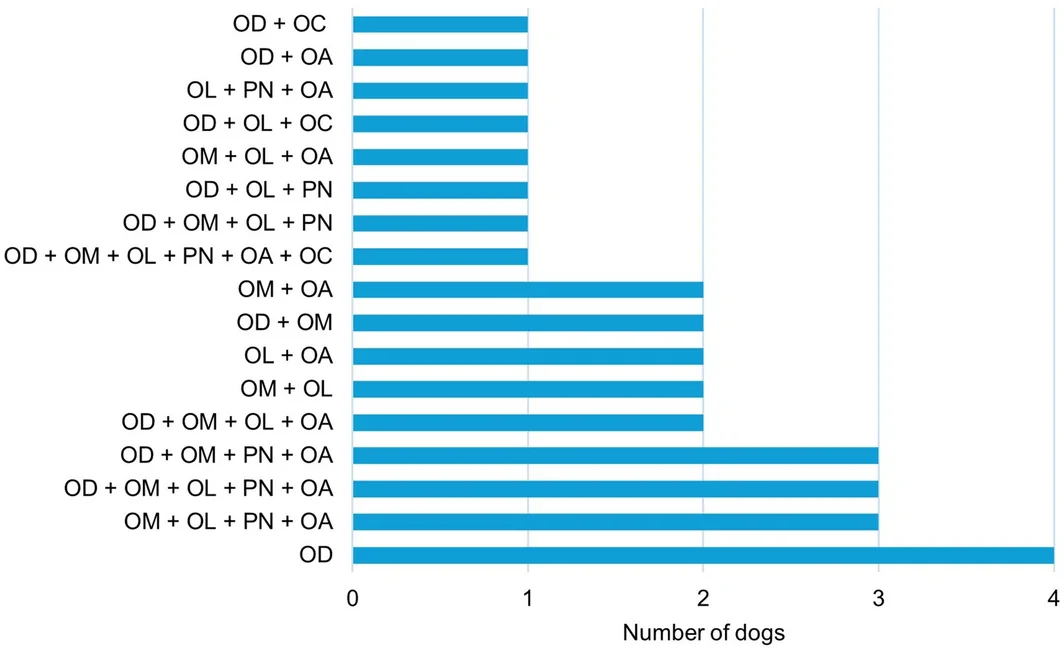
Claw abnormalities observed at initial presentation in 31 dogs with symmetrical lupoid onychodystrophy. OA, onychalgia; OC, onychoschizia; OD, onychodystrophy; OL, onycholysis; OM, onychomadesis; PN, paronychia.

**TABLE 1:** Claw fold cytological findings reported for 16 dogs with symmetrical lupoid onychodystrophy.

Dog	Negative	Cocci	Rods	Yeast
1		x	x	
2		x		x
3	x			
4		x	x	
5	x			
6		x	x	
10		x		
12		x		
13		x		
18	x			
20		x		
21	x			
22		x	x	
23		x		
25	x			
31	x			

In 26 of 31 (83.87%) dogs, information about the number of paws affected was recorded. All four paws were affected in 19 of 26 (73.08%) dogs, three paws in 4 of 26 (15.38%), two paws in 2 of 26 (7.69%) and one paw in 1 of 26 (3.84%). Of 26 dogs, 25 (92.3%) presented with clinical signs of SLO in the right front paw and 25 (92.3%) in the left front paw, followed by 23 (88.46%) in the right hind paw and 20 (76.92%) in the left hind paw. In 26 of 31 dogs (83.87%), the number of claws affected was described in the medical records; 8 of 26 (30.76%) dogs' records mentioned multiple claws affected with no specific number reported, and 18 of 26 (69.23%) dogs had the number of affected claws described per paw (Table S4).

Regarding diagnostic methods, a clinical diagnosis was achieved in 30 of 31 (96.77%) cases, based on the patient's medical history and clinical examination. One of these 30 dogs had a prior biopsy sample, yet the histopathological results were inconclusive. SLO was diagnosed based on clinical evidence. Only one (of 31, 3.22%) dog had a definitive diagnosis obtained on histopathological evaluation of an excisional biopsy sample of the right front dewclaw.

Initial treatment protocols varied among SLO patients and included combinations of immunomodulatory agents, antibiotics, analgesics and topical therapies. The most frequently prescribed treatments were tetracyclines (24 of 30, 80%), followed by niacinamide (23 of 30, 76.66%), EFAs (23 of 30, 76.66%), vitamin E (17 of 30, 56.66%), pentoxifylline (17 of 30, 56.66%), biotin (10 of 30, 33.33%) and prednisone (3/30, 10%). The therapy combinations are listed in Figure 4.

**FIGURE 4:**
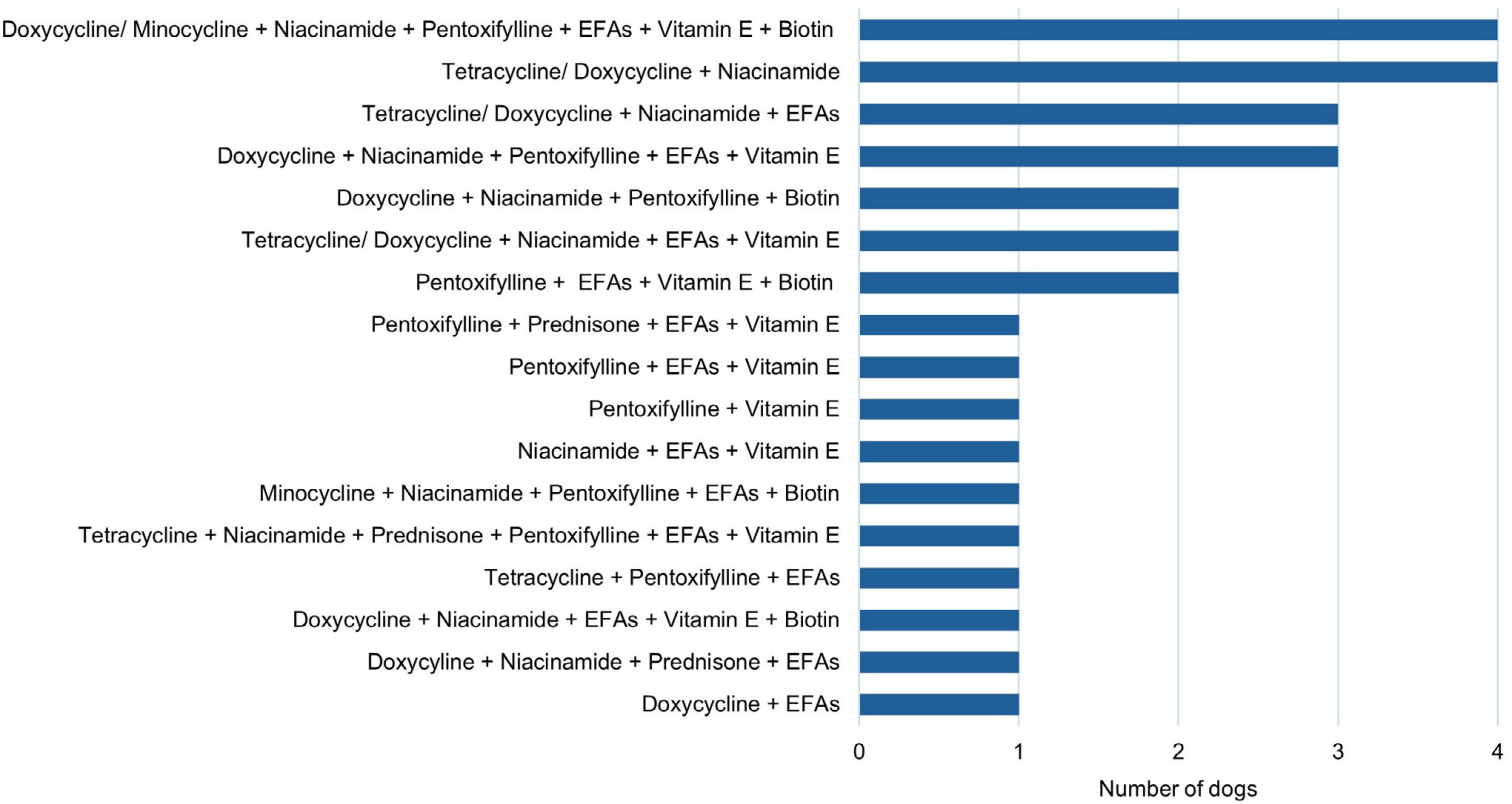
Treatment protocols recommended for dogs with symmetrical lupoid onychodystrophy at their initial appointment. EFAs, essential fatty acids.

A strict diet trial with a hydrolysed diet to investigate possible food hypersensitivity associated with SLO was recommended for two dogs yet declined by the owners. Four dogs had already gone through a diet trial as part of the work‐up for their previously diagnosed allergic disease, yet the pruritus did not subside with the diet change and they were not being fed a hydrolysed diet at the time of the SLO diagnosis. Four other dogs were instructed to change their diets to one with a higher concentration of EFAs yet did not undergo a strict diet trial.

Different systemic antibiotics and topical therapies were prescribed to treat the secondary bacterial infection of the claw fold (Tables S5 and S6). They were not included in the descriptive analysis as these medications were not prescribed for the treatment of the primary SLO disease.

Seven (of 31, 22.58%) dogs were lost to follow‐up after their initial SLO diagnosis and were not rechecked. The first recheck visit occurred within 1 month after SLO diagnosis for 11 of 24 (45.83%) dogs, between 1 and 3 months for 10 of 24 (41.66%) and after 3 months for 3 of 24 (12.5%) dogs. At the time of the first recheck, 15 of 24 (62.5%) of the dogs had SLO clinical signs considered well‐controlled, 7 of 24 (29.16%) satisfactorily controlled and 2 of 24 (8.33%) were not controlled. Complete remission of SLO was not observed in any studied dog. Individual information about initial treatment plans, timeframe of the first recheck and response to treatment is presented in Table S6.

Follow‐up information after the first recheck was available for 18 of 24 (75%) dogs receiving long‐term maintenance therapy. SLO was controlled in 13 of 18 (72.22%) dogs and uncontrolled in five of 18 (27.78%) dogs. Ten (of 15, 66.66%) dogs that presented well‐controlled disease at the first recheck remained with the disease controlled during maintenance (dogs 2, 5, 7, 13, 15, 16, 18, 19, 20 and 21). Dogs 3 and 30 were satisfactorily controlled at the first recheck and achieved control of SLO clinical signs during follow‐up. Dog 22 was not controlled at the first recheck and achieved control of SLO clinical signs during follow‐up. Dogs 12, 14, 26 and 29 that were satisfactorily controlled at their first recheck, flared up during follow‐up and were considered uncontrolled. Dog 25, that was not controlled at the first recheck, remained uncontrolled during follow‐up.

Of the 13 dogs with controlled SLO after the first recheck, the time when SLO control was achieved was available for 12 of 13 (92.30%) dogs. For 2 of 12 (16.66%) dogs, maintenance was achieved in 1–3 months after treatment was initiated; for 5 of 12 (41.67%) in 3–6 months and for 5 of 12 (41.67%) in 6–12 months.

Treatment protocols used for maintenance therapy for the 13 dogs with the disease controlled after the first recheck are presented in Figure 5. EFAs were included in the long‐term maintenance therapy for 14 of 18 (77.77%) dogs and 12 of these dogs (of 13, 92.30%) were controlled cases. Of 18 dogs, maintenance therapy treatment used vitamin E for 12 (66.66%), pentoxifylline for 10 (55.55%), biotin for 7 (38.88%), tetracyclines for 6 (33.33%), niacinamide for 5 (27.77%) and prednisone for 1 (5.55%). Of the 13 dogs with controlled SLO, three (dogs 18, 20 and 21) were maintained with pentoxifylline, EFAs and vitamin E and two (dogs 3 and 13) only with supplements (EFAs, biotin and vitamin E). One dog (Dog 2) received a combination of ciclosporin, EFAs, biotin and vitamin E; and another dog (Dog 16) was maintained with ciclosporin, pentoxifylline, EFAs, biotin and vitamin E.

**FIGURE 5:**
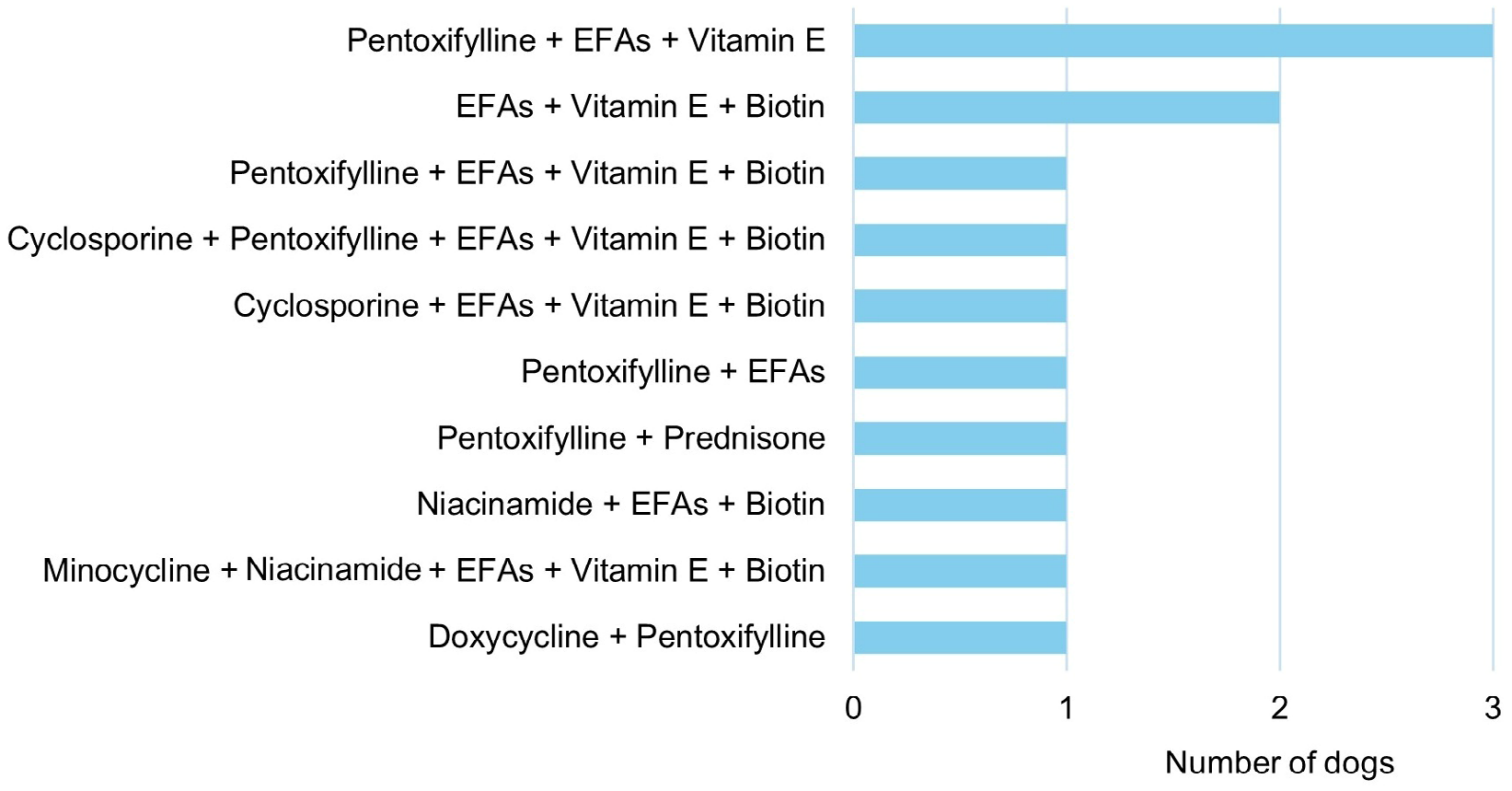
Long‐term maintenance treatment administered to dogs with well‐controlled symmetrical lupoid onychodystrophy after the first recheck visit. EFAs, essential fatty acids.

For the 18 dogs with follow‐up information available, 14 (77.78%) dogs were monitored for > 1 year, 3 (16.67%) for 6–12 months and 1 (5.56%) for 3–6 months. Data from maintenance therapy for each dog, clinical presentation during follow‐up, timeframe to achieve disease control, long‐term therapy and follow‐up period are presented in Table S7.

The dosage and frequency of treatment prescribed for dogs with SLO are described in Table S8. The correlation between dose and response to treatment of different therapies was beyond the scope of this descriptive study.

## 4 Discussion

Since SLO was first reported in 1992 by Scott and Miller [12], there have been few studies describing the disease and affected dogs. Current practice regarding the diagnosis and management of dogs with SLO and suspected different aetiological aspects of the disease has been providing further insights into this condition.

Consistent with previous studies, the dogs' age at the time of SLO onset ranged from young to senior [3, 4, 5, 6, 10, 13, 14] and two of the breeds previously indicated to be pre‐disposed, German shepherd dogs [4] and bearded collies [5, 15, 16], also were seen in this case series. Although SLO has been reported most frequently in large breed dogs, a few small and medium breed dogs were represented in this study, which may reflect the popularity of some breeds in different geographical locations. It would be interesting to investigate any potential association between the size of the dog or genetic background and disease development, or if other factors different than size or breed such as environment, diet, vaccination or other concurrent diseases could possibly be involved in triggering SLO. Owing to the retrospective nature of this study, the information was not available in the patients' records and could not be analysed.

The terms symmetrical lupoid onychomadesis or just symmetrical onychomadesis have been previously proposed for this condition as some dogs may show a sudden onset of onychomadesis without other clinical signs [5, 6, 17]. Interestingly, not every dog in this study presented with onychodystrophy or onychomadesis, reinforcing the clinical variability of the condition, the possibility of different aetiologies and the importance of a detailed physical examination.

Differential diagnoses that should be considered for SLO include trauma, neoplasia, leishmaniasis, dermatophytosis, food‐induced atopic dermatitis and immune‐mediated diseases such as vasculitis, systemic lupus erythematosus and cutaneous drug reaction [4, 6, 10]. Trauma and neoplasia are most often observed affecting only one claw. One of the dogs included in the study presented with clinical signs in only one paw yet two claws on the second and fourth digits were affected, and trauma and neoplasia were considered less likely differentials. Other infectious and immune‐mediated diseases normally present with different cutaneous and/or systemic signs, not being limited to the claws alone. One dog diagnosed with SLO also had a prior diagnosis of discoid lupus erythematosus (Dog 7, a Labrador retriever) and another a prior diagnosis of systemic lupus erythematosus (Dog 16, a German shepherd dog). It is not known if there is an association between the different lupus diseases and SLO, or if in the latter case the claw disease was a consequence of the systemic lupus instead of an SLO case. Dogs with leishmaniasis can present with onychogryphosis (excessive growth and abnormal curvature of multiple or all claws); therefore, this parasitic disease should always be considered in endemic areas and when these findings are observed [18]. The possibility of food allergies being involved in the development of SLO has been noted previously [4]. We could not retrieve any information about strict diet trials and outcomes from the dogs' records, yet the recommendation can be considered when discussing the management of dogs with SLO.

Biopsy collection and histopathological examination of claws have been recommended as the definitive diagnostic approach for SLO, which either involves an amputation of the third phalanx of the digit or the dewclaw if affected [8, 19], or an onychobiopsy sample without onychectomy that requires familiarity with the technique [9]. More recently a clinical diagnosis of SLO based on the patient's signalment, history, and clinical signs has been encouraged. Over the study's 20 year span, 16 clinicians were involved in the dogs' medical care, and only one dog had a biopsy taken to confirm the diagnosis, reinforcing that the clinical diagnosis of SLO is widely accepted by many veterinary surgeons.

In most dogs that were presented with paronychia and had cytological information in their records, a bacterial infection was diagnosed. In one dog a yeast infection was concomitantly identified. These findings support the cytological examination of the affected claw fold lesions as part of the dermatological work‐up and corroboration with previous observations [10].

Dogs received multimodal therapy, with different medications and supplements at variable dosing and frequency, making it difficult to attribute success to one treatment. A commonly prescribed protocol was doxycycline (or tetracycline or minocycline) and niacinamide which historically has been considered safe and effective in controlling the signs of SLO in some dogs [3, 10, 20]. However, the use of antibiotics for a disease that is not primarily bacterial should be avoided to comply with good antimicrobial stewardship practice.

Our findings show that EFAs seem to play an important role in both initial and maintenance treatment of dogs with SLO, corroborating other studies in which dogs were treated with combinations of drugs and EFAs, and showed good‐to‐excellent clinical response within a few months [8, 13], and in which some dogs flared up when monotherapy with EFAs as maintenance was withdrawn [13]. However, when only using EFAs as the initial and maintenance therapies for SLO, clinical improvement may not be observed [19].

Vitamin E is often considered for immune‐mediated and inflammatory conditions in dogs. It has previously been documented in combination with other medications [5] and with EFAs for SLO [3, 6, 10, 13, 14]. In the same way, it was used in the current study as a supplementary therapy, yet its role in clinical improvement cannot be evaluated.

Pentoxifylline has been consistently used as the only treatment for SLO or in combination with other medications and supplements, with variable clinical response [3, 21]. In some cases, it seemed to promote clinical improvement in dogs that did not respond to doxycycline + niacinamide and EFAs [3]. In our study, pentoxifylline was not used as a single treatment for SLO, rather added to different protocols for the initial and maintenance therapies; it was part of the maintenance protocol prescribed to about one‐third of the dogs that were clinically controlled.

A more recent study compared ciclosporin given with a diet rich in EFAs to only EFAs for the treatment of SLO in dogs and showed no difference in clinical improvement between groups [14]. In the current study ciclosporin was administered with other medications in a maintenance protocol to two clinically controlled dogs. Its use may not always be necessary, yet if given it is a safe, well‐tolerated treatment that can be considered a replacement for the tetracyclines + niacinamide combination, with a possible similar clinical outcome.

Lastly, a combination of biotin and zinc has been used in two dogs with no effect over the claw disease [10]. The use of these supplements or zinc as a single agent was not noted in our study population.

Despite the inherent limitations of a retrospective study such as inconsistent records, different treatment protocols, variable clinical presentation and outcome reports, we observed that the distinctive presentation of SLO supports its clinical diagnosis. The condition can be diagnosed in any dog regardless of age or breed. Different treatment protocols seem to be effective in controlling the condition, although spontaneous remission has also been described previously. EFAs stood out as support therapy and should be considered in combination with other supplements and/or medications such as pentoxifylline and ciclosporin for the treatment of SLO in dogs.

## Author Contributions

**Jessica R. Calesso:** conceptualization, investigation, methodology, writing – original draft, project administration, data curation, writing – review and editing, formal analysis. **Stephanie R. Bruner:** writing – review and editing, methodology, writing – original draft. **Adriane P. C. V. Bicalho:** supervision, project administration. **Clarissa P. Souza:** conceptualization, methodology, writing – original draft, writing – review and editing, data curation, supervision, resources, formal analysis.

## Funding

Self‐funded.

## Conflicts of Interest

The authors declare no conflicts of interest.

## Supporting information


**Table S1:** Signalment and age at diagnosis of 31 dogs with symmetrical lupoid onychodystrophy.

**Table S2:** Concurrent diseases in 21 of 31 dogs presented with symmetrical lupoid onychodystrophy.

**Table S3:** Frequency of claw abnormalities observed in 31 dogs with symmetrical lupoid onychodystrophy.

**Table S4:** Number of claws affected on each paw on 31 dogs with symmetrical lupoid onychodystrophy.

**Table S5:** Systemic antibiotics prescribed at the initial visit to address the secondary bacterial infection likely to have been caused by symmetrical lupoid onychodystrophy.

**Table S6:** Treatment prescribed at the first visit, timeframe of the first recheck, and response to initial treatment for 31 dogs with symmetrical lupoid onychodystrophy.

**Table S7:** Clinical outcome and timeframe to achieve control after initial therapy, maintenance therapy and follow‐up duration for 31 dogs with symmetrical lupoid onychodystrophy.

**Table S8:** Dosages of medications and supplements prescribed for 31 dogs with symmetrical lupoid onychodystrophy.

## Data Availability

The data that supports the findings of this study are available in the Supporting Information of this article.
