# Supplementary material for: Symmetrical Lupoid Onychodystrophy in Review: Lessons Learned From 31 Cases
Source: Vet Dermatol. 2026 Aug 6;37(6):941–7. doi: 10.1111/vde.70114 (PMC13751061; doi:10.1111/vde.70114)
Supplement: Supplementary file 1 [file VDE-37-941-s001.docx]

**TABLE S8.** Dosages of medications and supplements prescribed for 31 dogs with symmetrical lupoid onychodystrophy.

| Treatment prescribed | Dose range  Min Max | | Average dose |
| --- | --- | --- | --- |
| Tetracycline | 9.84 mg/kg | 37.59 mg/kg | 23.05 mg/kg |
| Doxycycline | 2.9 mg/kg | 10 mg/kg | 5.9 mg/kg |
| Minocycline | 5.9 mg/kg | 6.65 mg/kg | 6.27 mg/kg |
| EFAs | 10.35 mg/kg | 143.51 mg/kg | 46.11 mg/kg |
| Biotin | 110.37 mcg/kg | 868.06 mcg/kg | 311.09 mcg/kg |
| Vitamin E | 7.76 IU/kg | 41.23 IU/kg | 15.62 IU/kg |
| Pentoxfylline | 8.8 mg/kg | 22.7 mg/kg | 16.75 mg/kg |
| Prednisone | 0.4 mg/kg | 2.1 mg/kg | 1.01 mg/kg |

EFAs, essential fatty acids.
