# Supplementary material for: Symmetrical Lupoid Onychodystrophy in Review: Lessons Learned From 31 Cases
Source: Vet Dermatol. 2026 Aug 6;37(6):941–7. doi: 10.1111/vde.70114 (PMC13751061; doi:10.1111/vde.70114)
Supplement: Supplementary file 2 [file VDE-37-941-s002.docx]

**TABLE S2.** Concurrent diseases in 21 of 31 dogs presented with symmetrical lupoid onychodystrophy.

| Concurrent diseases | Dog numbers |
| --- | --- |
| Atopic dermatitis (possibly environmentally induced) | 5, 15, 25 |
| Atopic dermatitis (possibly food-induced) | 29 |
| Interdigital cysts / furunculosis | 6, 12 |
| Discoid lupus erythematous | 7 |
| Systemic lupus erythematous | 16 |
| Metacarpal fistula | 12 |
| Dermatophytosis | 15 |
| Osteoarthritis | 13, 14, 15 |
| Hypothyroidism | 2; 14; 16 |
| Valvular disease | 11; 27 |
| Ventricular arrhythmia | 3 |
| Dilated cardiomyopathy | 28 |
| Seizures | 10; 26; 29 |
| Bilateral laryngeal paralysis | 19 |
| Intervertebral disc disease | 3 |
| Hepatopathy | 9; 23; 24 |
| Chronic kidney disease | 27 |
| Chronic superficial keratitis | 19 |
| Bilateral cataracts | 15 |
