# Supplementary material for: Symmetrical Lupoid Onychodystrophy in Review: Lessons Learned From 31 Cases
Source: Vet Dermatol. 2026 Aug 6;37(6):941–7. doi: 10.1111/vde.70114 (PMC13751061; doi:10.1111/vde.70114)
Supplement: Supplementary file 3 [file VDE-37-941-s003.docx]

**TABLE S4.** Number of claws affected on each paw on 31 dogs with symmetrical lupoid onychodystrophy.

| Dog | Claws affected on each paw |
| --- | --- |
| 1 | LF: 2 |
| 2 | RF: 4, LF: 4, RH: 4, LH: 4 |
| 3 | RF: 1, LF: 1, RH:1, LH:2 |
| 4 | RF: 3, LF: 2, RH: 3, LH: 2 |
| 5 | RF: 3, LF: 3, RH: 1, LH: 1 |
| 6 | RF: 1, LF:1, RH: 1 |
| 7 | RF: 1, LF: 3, RH: 3, LH: 3 |
| 8 | Multiple |
| 9 | RF: 4, LF: 4, RH: 4, LH: 4 |
| 10 | RF: 5, LF: 5, RH: 4, LH: 4 |
| 11 | Multiple |
| 12 | RF: 3, LF: 2, RH: 4, LH: 4 |
| 13 | Multiple |
| 14 | RF: 1, LF: 1, RH: 1 |
| 15 | All claws |
| 16 | Multiple |
| 17 | RF: 3, LF: 2, RH: 1, LH: 2 |
| 18 | RF: 3, LF: 2, RH: 2, 1 |
| 19 | RF: 1, LF: 1 |
| 20 | RF: 1, RH: 1, LH: 1 |
| 21 | RF: 3, LF: 2, RH: 2, LH: 2 |
| 22 | Multiple |
| 23 | Multiple |
| 24 | RF: 1, LF:1 |
| 25 | Not reported |
| 26 | Not reported |
| 27 | Not reported |
| 28 | Not reported |
| 29 | All claws |
| 30 | Not reported |
| 31 | Multiple |

RF, right front; LF, left front; RH, right hind; LH, left hind.
