# Supplementary material for: Symmetrical Lupoid Onychodystrophy in Review: Lessons Learned From 31 Cases
Source: Vet Dermatol. 2026 Aug 6;37(6):941–7. doi: 10.1111/vde.70114 (PMC13751061; doi:10.1111/vde.70114)
Supplement: Supplementary file 4 [file VDE-37-941-s004.docx]

**TABLE S6.** Treatment prescribed at the first visit, timeframe of the first recheck, and response to initial treatment for 31 dogs with symmetrical lupoid onychodystrophy.

|  | Initial treatment prescribed | | | | | | | | | | | | | | Timeframe of first recheck (months) | | | | Clinical outcome after initial treatment | | | |
| --- | --- | --- | --- | --- | --- | --- | --- | --- | --- | --- | --- | --- | --- | --- | --- | --- | --- | --- | --- | --- | --- | --- |
|  | *Systemic treatment* | | | | | | | | | *Topical treatment* | *Pain control* | | |  | | | | | | | | |
| Dog | T | D | M | N | EFAs | B | Vit E | Pen | Pred | * | G | Tr | C | <1 | >1-3 | >3 | No follow-up | WC | | SC | NC |  |
| 1 | x |  |  | x | x |  |  |  |  | x |  |  | x | x |  |  |  | x | |  |  |  |
| 2 |  | x |  | x | x | x | x |  |  | x |  |  |  | x |  |  |  | x | |  |  |  |
| 3 |  |  |  |  | x | x | x | x |  |  |  |  |  |  | x |  |  |  | | x |  |  |
| 4 |  | x |  | x | x |  | x | x |  | x |  |  |  |  | x |  |  | x | |  |  |  |
| 5 |  | x |  | x | x |  |  |  | x |  |  | x |  |  | x |  |  | x | |  |  |  |
| 6 |  | x |  | x | x | x | x | x |  |  |  |  |  |  | x |  |  |  | | x |  |  |
| 7 |  | x |  | x | x | x | x | x |  |  |  |  |  |  |  | x |  | x | |  |  |  |
| 8 | x |  |  | x |  |  |  |  |  |  |  |  |  |  | x |  |  | x | |  |  |  |
| 9 | x |  |  | x | x |  |  |  |  |  |  |  |  |  |  |  | x |  | |  |  |  |
| 10 |  | x |  | x |  | x |  | x |  |  |  |  |  | x |  |  |  | x | |  |  |  |
| 11 |  | x |  | x |  |  |  |  |  |  |  |  |  |  |  |  | x |  | |  |  |  |
| 12 |  | x |  | x | x |  | x |  |  | x |  | x |  |  |  | x |  |  | | x |  |  |
| 13 |  |  |  |  | x | x | x | x |  |  |  |  |  | x |  |  |  | x | |  |  |  |
| 14 |  | x |  | x |  | x |  | x |  |  |  |  | x | x |  |  |  |  | | x |  |  |
| 15 |  |  | x | x | x | x |  | x |  |  |  |  |  | x |  |  |  | x | |  |  |  |
| 16 |  |  | x | x | x | x | x | x |  |  |  |  |  |  | x |  |  | x | |  |  |  |
| 17 |  |  |  |  | x |  | x | x |  |  |  |  |  |  |  |  | x |  | |  |  |  |
| 18 |  | x |  | x | x | x | x | x |  |  |  |  |  |  | x |  |  | x | |  |  |  |
| 19 |  | x |  | x | x |  | x | x |  |  | x |  |  |  | x |  |  | x | |  |  |  |
| 20 |  |  |  |  |  |  | x | x |  |  |  |  |  | x |  |  |  | x | |  |  |  |
| 21 |  |  |  |  | x |  | x | x | x | x | x |  |  | x |  |  |  | x | |  |  |  |
| 22 |  | x |  | x | x |  | x | x |  |  | x |  |  |  | x |  |  |  | |  | x |  |
| 23 | x |  |  |  | x |  |  | x |  | x |  | x |  |  |  |  | x |  | |  |  |  |
| 24 | x |  |  | x | x |  | x | x | x |  |  |  |  |  |  |  | x |  | |  |  |  |
| 25 | x |  |  | x |  |  |  |  |  |  |  |  |  | x |  |  |  |  | |  | x |  |
| 26 | x |  |  | x |  |  |  |  |  |  |  |  |  | x |  |  |  |  | | x |  |  |
| 27 |  | x |  | x | x |  |  |  |  |  |  |  |  |  |  |  | x |  | |  |  |  |
| 28 | x |  |  | x | x |  | x |  |  | x |  |  |  |  |  |  | x |  | |  |  |  |
| 29 |  | x |  |  | x |  |  |  |  |  |  |  |  | x |  |  |  |  | | x |  |  |
| 30 |  |  |  |  |  |  |  |  |  |  |  |  |  |  | x |  |  |  | | x |  |  |
| 31 |  |  |  | x | x |  | x |  |  |  |  |  |  |  |  | x |  | x | |  |  |  |

T, tetracycline; D, doxycycline; M, minocycline; N, niacinamide; EFAs, essential fatty acids; B, biotin; Vit E, vitamin E; Pen, pentoxifylline; Pred, prednisone; G, gabapentin; Tr, tramadol; C, carprofen; WC, well-controlled; SC, satisfactorily controlled; NC, not controlled.

*Topical medications included: diluted antiseptic solutions for foot soak, such as chlorhexidine (dogs 1, 4, 12 and 23), gentamicin (Dog 2) and bleach (Dog 21); over the counter antiseptic spray solution with chlorhexidine (Dog 28); and gentamicin 0.1% cream (Dog 1).
