# Supplementary material for: Symmetrical Lupoid Onychodystrophy in Review: Lessons Learned From 31 Cases
Source: Vet Dermatol. 2026 Aug 6;37(6):941–7. doi: 10.1111/vde.70114 (PMC13751061; doi:10.1111/vde.70114)
Supplement: Supplementary file 5 [file VDE-37-941-s005.docx]

**TABLE S3.** Frequency of claw abnormalities observed in 31 dogs with symmetrical lupoid onychodystrophy.

| Clinical findings | Number of dogs | Proportion of dogs |
| --- | --- | --- |
| Onychodystrophy | 20 | 64.51% |
| Onychomadesis | 20 | 64.51% |
| Onychalgia | 19 | 61.29% |
| Onycholysis | 18 | 58.06% |
| Paronychia | 13 | 41.93% |
| Onychoschizia | 3 | 9.67% |
