# Supplementary material for: Symmetrical Lupoid Onychodystrophy in Review: Lessons Learned From 31 Cases
Source: Vet Dermatol. 2026 Aug 6;37(6):941–7. doi: 10.1111/vde.70114 (PMC13751061; doi:10.1111/vde.70114)
Supplement: Supplementary file 6 [file VDE-37-941-s006.docx]

**TABLE S5.** Systemic antibiotics prescribed at the initial visit to address the secondary bacterial infection likely to have been caused by symmetrical lupoid onychodystrophy.

| Dog | Antibiotic | Dose (mg/kg) | Frequency |
| --- | --- | --- | --- |
| 1 | Amoxicillin /Clavulanic acid | 20 | BID |
| 2 | Chloramphenicol | 42 | TID |
| 12 | Cephalexin | 23.8 | BID |
| 13 | Clindamycin | 7.5 | BID |
| 14 | Cefpodoxime | 4.4 | SID |
| 19 | Cephalexin | 31 | BID |
| 20 | Clindamycin | 9.4 | BID |
| 23 | Cefpodoxime | 5.3 | SID |

BID, every 12 h; TID, every 8 h; SID, every 24 h.
