# Supplementary material for: Symmetrical Lupoid Onychodystrophy in Review: Lessons Learned From 31 Cases
Source: Vet Dermatol. 2026 Aug 6;37(6):941–7. doi: 10.1111/vde.70114 (PMC13751061; doi:10.1111/vde.70114)
Supplement: Supplementary file 7 [file VDE-37-941-s007.docx]

**TABLE S7.** Clinical outcome and timeframe to achieve control after initial therapy, maintenance therapy and follow-up duration for 31 dogs with symmetrical lupoid onychodystrophy.

|  | Clinical outcome on first rechecks | | | Timeframe to achieve disease control (months) | | | | | | Maintenance therapy | | | | | | | | | | | Follow-up duration (months) | | | |  |
| --- | --- | --- | --- | --- | --- | --- | --- | --- | --- | --- | --- | --- | --- | --- | --- | --- | --- | --- | --- | --- | --- | --- | --- | --- | --- |
| Dog | C | U | No follow-up | <1 | >1–3 | >3–6 | >6–12 | T | D | | M | N | EFAs | B | Vit E | Pen | Pred | Topical***** | Ci | <3 | | >3–6 | >6–12 | >12 | |
| 1 |  |  | x |  |  |  |  |  |  | |  |  |  |  |  |  |  |  |  |  | |  |  |  | |
| 2 | x |  |  |  |  | x |  |  |  | |  |  | x | x | x |  |  | x | x |  | |  |  | x | |
| 3 | x |  |  |  |  |  | x |  |  | |  |  | x | x | x |  |  |  |  |  | |  |  | x | |
| 4 |  |  | x |  |  |  |  |  |  | |  |  |  |  |  |  |  |  |  |  | |  |  |  | |
| 5 | x |  |  |  |  | x |  |  |  | |  |  |  |  |  | x | x |  |  |  | |  |  | x | |
| 6 |  |  | x |  |  |  |  |  |  | |  |  |  |  |  |  |  |  |  |  | |  |  |  | |
| 7 | x |  |  |  |  | x |  |  |  | | x | x | x | x | x |  |  | x |  |  | |  | x |  | |
| 8 |  |  | x |  |  |  |  |  |  | |  |  |  |  |  |  |  |  |  |  | |  |  |  | |
| 9 |  |  | x |  |  |  |  |  |  | |  |  |  |  |  |  |  |  |  |  | |  |  |  | |
| 10 |  |  | x |  |  |  |  |  |  | |  |  |  |  |  |  |  |  |  |  | |  |  |  | |
| 11 |  |  | x |  |  |  |  |  |  | |  |  |  |  |  |  |  |  |  |  | |  |  |  | |
| 12 |  | x |  |  |  |  |  |  | x | |  | x | x |  | x |  |  | x |  |  | |  |  | x | |
| 13 | x |  |  |  | x |  |  |  |  | |  |  | x | x | x |  |  |  |  |  | |  |  | x | |
| 14 |  | x |  |  |  |  |  |  |  | |  |  |  |  | x | x |  |  |  |  | |  |  | x | |
| 15 | x |  |  |  |  |  | x |  |  | |  | x | x | x |  |  |  |  |  |  | |  |  | x | |
| 16 | x |  |  |  |  |  | x |  |  | |  |  | x | x | x | x |  |  | x |  | |  |  | x | |
| 17 |  |  | x |  |  |  |  |  |  | |  |  |  |  |  |  |  |  |  |  | |  |  |  | |
| 18 | x |  |  |  |  | x |  |  |  | |  |  | x |  | x | x |  |  |  |  | |  |  | x | |
| 19 | x |  |  |  |  |  | x |  | x | |  |  | x |  |  | x |  |  |  |  | |  |  | x | |
| 20 | x |  |  |  |  | x |  |  |  | |  |  | x |  | x | x |  |  |  |  | |  |  | x | |
| 21 | x |  |  |  | x |  |  |  |  | |  |  | x |  | x | x |  |  |  |  | |  | x |  | |
| 22* | x |  |  |  |  |  | x |  |  | |  |  | x | x | x | x |  |  |  |  | |  | x |  | |
| 23 |  |  | x |  |  |  |  |  |  | |  |  |  |  |  |  |  |  |  |  | |  |  |  | |
| 24 |  |  | x |  |  |  |  |  |  | |  |  |  |  |  |  |  |  |  |  | |  |  |  | |
| 25 |  | x |  |  |  |  |  | x |  | |  | x |  |  | x |  |  | x |  |  | |  |  | x | |
| 26 |  | x |  |  |  |  |  |  | x | |  |  |  |  |  |  |  | x |  |  | | x |  |  | |
| 27 |  |  | x |  |  |  |  |  |  | |  |  |  |  |  |  |  |  |  |  | |  |  |  | |
| 28 |  |  | x |  |  |  |  |  |  | |  |  |  |  |  |  |  |  |  |  | |  |  |  | |
| 29 |  | x |  |  |  |  |  | x |  | |  | x | x |  |  | x |  |  |  |  | |  |  | x | |
| 30 | x |  |  |  |  |  |  |  |  | |  |  | x |  |  | x |  |  |  |  | |  |  | x | |
| 31 |  |  | x |  |  |  |  |  |  | |  |  |  |  |  |  |  |  |  |  | |  |  |  | |

C, controlled; U, uncontrolled; T, tetracycline; D, doxycycline; M, minocycline; N, niacinamide; EFAs, essential fatty acids; B, biotin; Vit E, vitamin E; Pen, pentoxifylline; Pred, prednisone; G, gabapentin; Ci, ciclosporin.

*Topical therapy included: triamcinolone acetonide spray (dogs 25 and 26); triamcinolone acetonide and gentamicin spray (Dog 2); mometasone ointment (Dog 2); diluted antiseptic solutions for foot soaking including chlorhexidine (Dog 4) and bleach (Dog 2); and chlorhexidine mousse (Dog 12).

*Dog 22 also was receiving gabapentin for pain control.
