# Supplementary material for: Symmetrical Lupoid Onychodystrophy in Review: Lessons Learned From 31 Cases
Source: Vet Dermatol. 2026 Aug 6;37(6):941–7. doi: 10.1111/vde.70114 (PMC13751061; doi:10.1111/vde.70114)
Supplement: Supplementary file 8 [file VDE-37-941-s008.docx]

**TABLE S1.** Signalment and age at diagnosis of 31 dogs with symmetrical lupoid onychodystrophy.

| Dog | Sex | Breed | Weight (kg) | Age at diagnosis |
| --- | --- | --- | --- | --- |
| 1 | FS | Bearded collie | 19.3 | 8y10m |
| 2 | MN | Labrador retriever | 35.2 | 11y1m |
| 3 | MN | Rhodesian ridgeback | 41,4 | 6y9m |
| 4 | MN | Mixed-breed | 38.5 | 11y6m |
| 5 | MN | American Eskimo dog | 18.5 | 6y3m |
| 6 | MN | Great Dane | 77.1 | 10y5m |
| 7 | FS | Labrador retriever | 34 | 4y10m |
| 8 | FS | Mixed-breed | 24.3 | 4y |
| 9 | MN | Scottish terrier | 13.3 | 4y9m |
| 10 | MN | Terrier-type mix | 5.8 | 13y8m |
| 11 | FS | Miniature schnauzer | 8.5 | 9y3m |
| 12 | FS | German shepherd dog | 42 | 7y1m |
| 13 | FS | Mixed-breed | 20 | 6y |
| 14 | MN | German shepherd dog | 45.3 | 5y4m |
| 15 | MN | Australian shepherd dog | 22.8 | 7y6m |
| 16 | MN | German shepherd dog | 33.5 | 6y |
| 17 | FS | Belgian Malinois | 29.7 | 7y |
| 18 | FS | Shetland sheepdog | 9.7 | 5y1m |
| 19 | FS | Greyhound | 31 | 9y7m |
| 20 | FS | Siberian husky mix | 33 | 10y4m |
| 21 | MN | German shepherd dog | 48.8 | 5y |
| 22 | MN | German shepherd dog | 51.5 | 6y5m |
| 23 | MN | Cairn terrier | 8.8 | 13y1m |
| 24 | MN | Pit bull-type | 18.5 | 3y |
| 25 | FS | Mixed-breed | 32.3 | 7y3m |
| 26 | MN | Australian shepherd dog | 25.4 | 2y7m |
| 27 | FS | Dachshund | 3.5 | 13y3m |
| 28 | MN | Great Dane | 50.8 | 8y |
| 29 | FS | Beagle | 12 | 7y2m |
| 30 | MN | Labrador retriever | 43.7 | Not available |
| 31 | MN | German shorthaired pointer | 40.8 | 1y2m |

FS, female spayed; MN, male neutered; y, year(s); m, month(s)
